# Typhoid Fever

**Published:** 1898-05

**Authors:** A. W. Toland

**Affiliations:** Chappell Hill, Texas


For the Texas Medical Journal.

### Typhoid Fever.

BY A. W. TOLAND, M. D., CHAPPELL HILL, TEXAS.

[Read before the Brenham Medical Society, April 5th, 1898.]

*Mr. President and Gentlemen of the Society:*

While pondering on the subject for today, I decided to select the one engaging my thoughts most at the present time, to-wit: Typhoid fever or Texas typhoid fever, for indeed, there is a very decided difference between this fever in Texas, both in the symptoms and prognosis, and as laid down in our text books.

While studying this subject I came across an article read by W. A. Adams, M. D., of Fort Worth, Texas, before the Chicago Medical Society, on November 17th, 1897, the subject being "The Phases of Contagio Infectious Diseases, as Observed in Texas, from those as Observed in Other Portions of the United States." Dr. Adams states that the materies morbi in Texas typhoid fever is the same as exists in the typical cases as found in other States, because he has had to do several autopsies on both the typical and atypical cases, and found the characteristic pathological lesions in the ileocæcal region the same, with which we are all familiar.

Then there is another class of practitioners, who take the ground that no typhoid fever exists in Texas. Physicians, too, of great experience and ability, take this position, on the premises of the absence of so many of the symptoms as described by our recognised authorities and not having had opportunity to do autopsies in their fatal cases.

My first experience with this fever in epidemic form, was in Caldwell, Texas, in the summer and fall of 1887, which some of you, no doubt, remember, also that it was very fatal, especially with those physicians who used antipyrine to reduce the temperature; but with practitioners who used aconite, veratrum, gelsemium, belladonna, tincture nux vomica and sulphite of soda, as the symptoms indicated, and let alone the antipyrine, the mortality was greatly lessened.

Nearly every physician had a different name for the fever, such as typhoid malarial, slow, mixed hybrid fever, etc. I finally wrote to Prof. Deering J. Roberts, M. D., being my professor on practice in the Nashville Medical College, for information, also giving a full description of the fever without an autopsy being done. His reply was unsatisfactory, he calling it "a new and strange fever in Texas." But now, since the erection of medical colleges and hospitals within our State, having advantages inferior to none in the South, we have learned, for a fact, that we do have "typhoid fever" in Texas, but in a less severe form and with a mortality amounting to almost nothing when properly treated.

As to the treatment, there has been a great change in the last decade. "The turpentine treatment," first introduced by Dr. George B. Wood as a remedy for threatened perforation, after having been misused as a specific for the fever throughout its course, still remains where he left it, the best remedy for this one condition, threatened perforation. The mineral acid treatment came next and was an improvement. Then came the iodine treatment.

Next Bartholow with carbolic acid and iodine, Brand with the cold baths and antiseptic treatment, while now we have the Woodbridge treatment. The great therapeutists of Europe are now turning their attention to the intestinal antiseptics. The typhoid germs or bacilli enter the body through the mouth in most cases, and the principal lesion is an inflammation of the intestinal glands. The typhoid bacilli are discharged in numbers in the stools of the typhoid patient, and to some extent in the urine, showing that the battle ground with the typhoid bacillus is in the intestinal tract. There are two specific indications for treatment to be found in every case, namely: keep the intestinal canal aseptic, and nourish. After these two specific indications the treatment will depend upon the symptoms of each individual case.

I will briefly report one of my most recent cases:

R. P., a little 7 year old white girl. I was called when she had been sick about one week with headache, aching of limbs, etc.; had some fever; temperature 103, and bowels tympanitic. Prescribed five half grain doses calomel, one hour apart, dry on tongue followed with water.

℞ Sulpho carbolate of zinc........................grs. xx

Ten granules each of

Codeine, grs. $\frac{1}{67}$,

Hyoscyamine, grs. $\frac{1}{1000}$,

Strychnine arsen., grs. $\frac{1}{84}$,

Water........................................q. s. ad. ℥iij

M. Sig.: Teaspoonful every two hours when awake. Gave high enema of one quart of warm water with a teaspoonful of tincture of asafœtida and ½ teaspoonful of spirits of turpentine to relieve accumulated gas, and rubbed abdomen with equal parts of turpentine and lard one or more times daily, as long as the bowels were tender, but was careful to rub gently and cover with warm flannel. Whenever fever reached 102° we ordered a three-grain zomokym powder every two hours until fever receded. We repeated the calomel in small doses, as indicated, until fever broke. Used the sponge bath every day one or more times. Gave nuclein by the mouth every day one or more times as needed. Gave the high enema when bowels were tympanitic and patient restless. Fever broke on the twenty-third day, and she has made a rapid convalescence.

